# ATTITUDES TOWARD ADVANCE DIRECTIVES AMONG OLDER SOUTHEAST ASIAN AMERICANS

**DOI:** 10.1093/geroni/igad104.0725

**Published:** 2023-12-21

**Authors:** Kaipeng Wang, Carson De Fries, My Ngoc To, Fei Sun, Bei Wu, Sue Levkoff

**Affiliations:** University of Denver, Denver, Colorado, United States; University of Denver, Denver, Colorado, United States; University of Denver, Denver, Colorado, United States; Michigan State University, East Lansing, Michigan, United States; New York University, New York City, New York, United States; University of South Carolina, Columbia, South Carolina, United States

## Abstract

Advance directives (ADs) are legal documents that enable individuals to communicate their end-of-life care preferences in advance. AD completion is associated with decreased likelihood of unwanted medical interventions, healthcare costs, and decisional burden for family members. Compared to older Americans in general, older Asian Americans are less likely to complete ADs. While research focusing on ADs among Asian Americans has grown, few have investigated attitudes toward ADs among older Southeast Asian Americans (SEAAs). The study addresses that gap by examining factors associated with attitude toward ADs among older SEAAs. Using convenience sampling from 12 community partners and snowball sampling, we recruited 251 participants aged 55 and older who self-identified as Vietnamese (n=113), Filipino (n=100), or Indonesian (n=38) Americans. Through online or in-person questionnaires, participants completed the Nolan and Bruder’s Advance Directive Attitude Survey, which measures attitudes toward ADs. Responses are rated on a 4-point Likert scale, with higher scores indicating more positive attitudes. In general, Indonesian (mean=3.40, SD=0.55) and Filipino (mean=3.40, SD=0.68) Americans expressed significantly more positive attitude toward ADs than Vietnamese (mean=3.15, SD=0.56) Americans. Results of ordinary least squares regression showed that family cohesion, expectation of intergenerational support, depression, and resilience were positively associated with attitude toward ADs, whereas death anxiety was negatively associated with attitude toward ADs. By enhancing understanding of SEAAs’ attitudes towards ADs, these results can inform culturally appropriate interventions to improve AD implementation among SEAAs and contribute to broader awareness of and access to end-of-life care services in this population.

